# A novel “Hammock method” for retrieval of large specimens after gastric endoscopic submucosal dissection using a dual-channel endoscope

**DOI:** 10.1055/a-2893-7076

**Published:** 2026-06-24

**Authors:** Shinji Yoneda, Yoshiki Tsujii, Ryotaro Uema, Hiromu Fukuda, Eiji Kimura, Yoshito Hayashi, Takahiro Kodama

**Affiliations:** 1 Department of Gastroenterology and Hepatology 38637 The University of Osaka Graduate School of Medicine Faculty of MedicineSuitaOsaka PrefectureJapan

A woman in her 60s was diagnosed with a 60-mm type 0-I early gastric cancer located on the greater curvature of the lower gastric body. Endoscopic submucosal dissection (ESD) was performed using a GIF-Q260J endoscope (Olympus Medical Systems, Tokyo, Japan) and a FlushKnife BT-S 2.5 mm (Fujifilm, Tokyo, Japan; Fig. 1 ). After successful *en bloc* resection, specimen retrieval was attempted using a standard retrieval net (18–30 mm; Olympus Medical Systems, Tokyo, Japan). Although most specimens could be placed within the net, resistance was encountered at the esophagogastric junction during withdrawal, and the specimen repeatedly dislodged with traction, raising concerns regarding specimen damage.

**Fig. 1:**
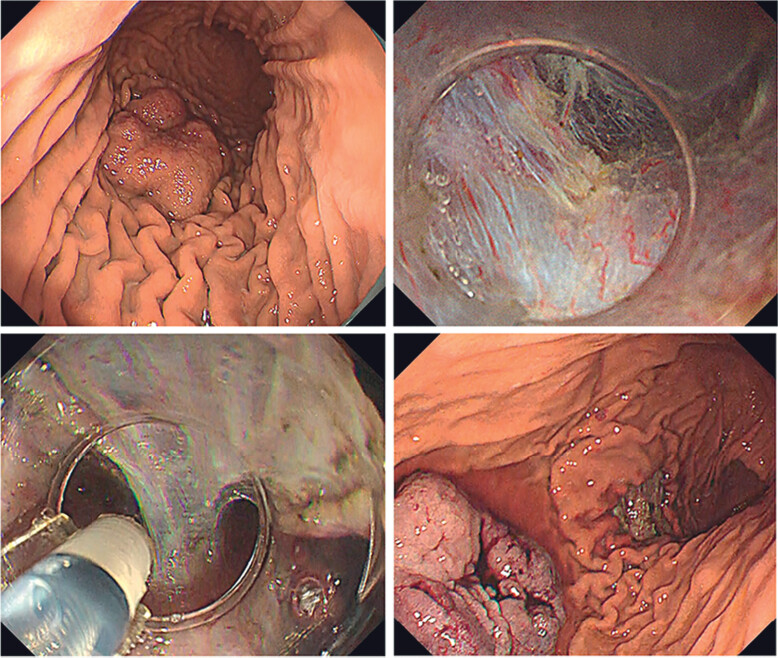
Endoscopic images of the lesion and endoscopic submucosal dissection.

Therefore, a retrieval technique using a dual-channel endoscope (GIF-2TQ260M; Olympus Medical Systems, Tokyo, Japan) was employed ( Fig. 2 and Video 1 ). A retrieval net and grasping forceps were inserted through separate working channels. The net was deployed beneath the specimen and its distal edge was grasped with forceps. Gently pulling the net into the endoscope channel folded it, maintaining an open configuration for hammock-like support. The endoscope and the net were withdrawn together. This technique is called the “Hammock method.” Mild resistance was encountered at the esophagogastric junction; however, the specimen remained stable. Post-retrieval endoscopy revealed no mucosal injury and the specimen showed no macroscopic damage. Histopathology demonstrated intramucosal carcinoma with negative margins and no lymphovascular involvement. The patient’s postoperative course was uneventful.

**Fig. 2:**
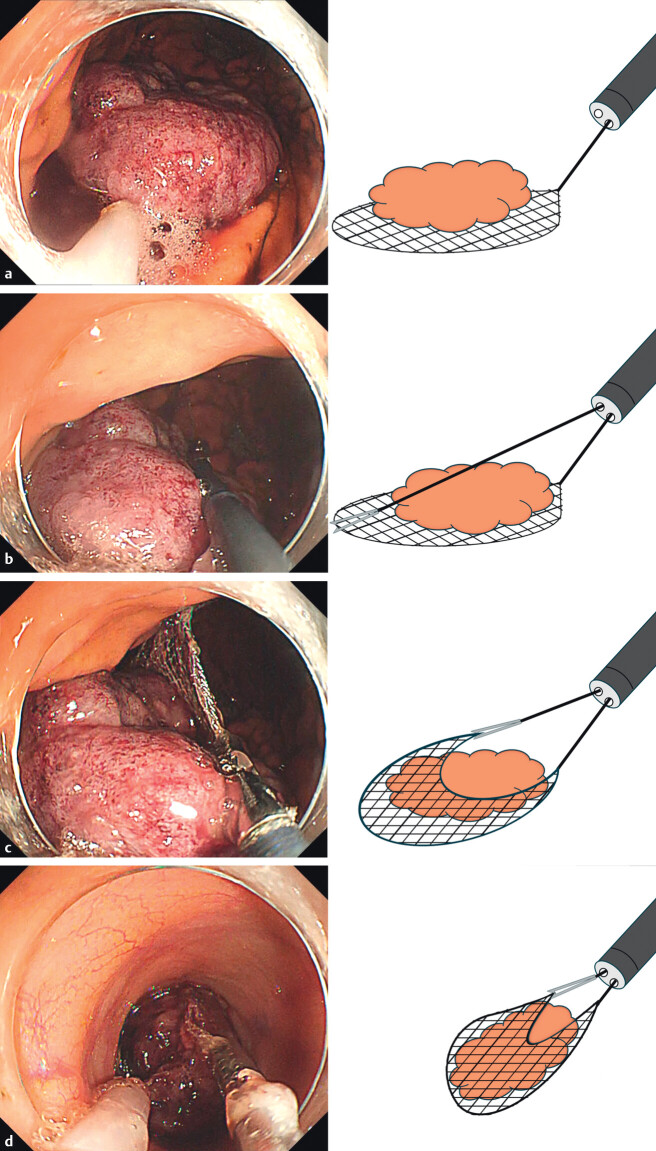
The retrieval technique using the Hammock method. ( **a** ) A retrieval net is deployed beneath the specimen. ( **b** ) The distal edge of the net is grasped using grasping forceps. ( **c** ) By gently pulling the grasped net into the endoscope channel, the net folds while maintaining its open configuration, allowing for hammock-like support. ( **d** ) The specimen is retrieved by withdrawing the endoscope and the net.

**Video 1** The “Hammock method,” using a dual-channel endoscope, support of large ESD specimens, facilitating safe retrieval.


Although various techniques exist for the retrieval of large ESD specimens, many require additional equipment. 1​ 2​ ​3​ The Hammock method uses standard devices with a dual-channel endoscope, offering a simple, practical option that may preserve specimen integrity as conventional net retrieval is insufficient.

Endoscopy_UCTN_Code_TTT_1AO_2AN

## References

[1] Tanaka S Toyonaga T East J Endoscopic retrieval method using a small grip-seal plastic bag for large colorectal resection specimens after endoscopic submucosal dissection Endoscopy 2010 42 E186 E187 10.1055/s-0029-1244168 02 20680918

[2] Nakazawa K Hirata Y Nakamura S Giant fecal stone recovered using a novel protective retrieval device Dig Endosc 2023 35 e55 e56 10.1111/den.14520 36776023

[3] Kobayashi N Kobara H Nishiyama N Newly developed endoscopic retrieval device: funnel-shaped overtube formed by air inflation-deflation Endoscopy 2023 55 E563 E565 10.1055/a-2040-3979 36958352 PMC10036206

